# Estimation of Uncertainties in the Global Distance Test (GDT_TS) for CASP Models

**DOI:** 10.1371/journal.pone.0154786

**Published:** 2016-05-05

**Authors:** Wenlin Li, R. Dustin Schaeffer, Zbyszek Otwinowski, Nick V. Grishin

**Affiliations:** 1 Howard Hughes Medical Institute, University of Texas Southwestern Medical Center, Dallas, Texas, 75390–9050, United States of America; 2 Department of Biochemistry and Department of Biophysics, University of Texas Southwestern Medical Center, Dallas, Texas, 75390–9050, United States of America; University of Michigan, UNITED STATES

## Abstract

The Critical Assessment of techniques for protein Structure Prediction (or CASP) is a community-wide blind test experiment to reveal the best accomplishments of structure modeling. Assessors have been using the Global Distance Test (GDT_TS) measure to quantify prediction performance since CASP3 in 1998. However, identifying significant score differences between close models is difficult because of the lack of uncertainty estimations for this measure. Here, we utilized the atomic fluctuations caused by structure flexibility to estimate the uncertainty of GDT_TS scores. Structures determined by nuclear magnetic resonance are deposited as ensembles of alternative conformers that reflect the structural flexibility, whereas standard X-ray refinement produces the static structure averaged over time and space for the dynamic ensembles. To recapitulate the structural heterogeneous ensemble in the crystal lattice, we performed time-averaged refinement for X-ray datasets to generate structural ensembles for our GDT_TS uncertainty analysis. Using those generated ensembles, our study demonstrates that the time-averaged refinements produced structure ensembles with better agreement with the experimental datasets than the averaged X-ray structures with B-factors. The uncertainty of the GDT_TS scores, quantified by their standard deviations (SDs), increases for scores lower than 50 and 70, with maximum SDs of 0.3 and 1.23 for X-ray and NMR structures, respectively. We also applied our procedure to the high accuracy version of GDT-based score and produced similar results with slightly higher SDs. To facilitate score comparisons by the community, we developed a user-friendly web server that produces structure ensembles for NMR and X-ray structures and is accessible at http://prodata.swmed.edu/SEnCS. Our work helps to identify the significance of GDT_TS score differences, as well as to provide structure ensembles for estimating SDs of any scores.

## Introduction

The Critical Assessment of techniques for protein Structure Prediction (or CASP) is a community-wide experiment to establish the capabilities and limitations of structure prediction methods, as well as to determine the progress of modeling methodologies [1]. Since CASP3 in 1998, assessors have been using the Global Distance Test (GDT_TS) score [2,3] in model evaluation due to its tolerance for partial structure segments that could create a large root mean square deviation (RMSD). The GDT algorithm uses the residue correspondence between the model and the target structure to search for optimal superpositions under selected distance cutoffs. The GDT_TS score reports an average of the maximum number of residues that can be superimposed under four distance cutoffs 1Å, 2Å, 4Å, and 8Å. Current GDT_TS comparisons produce a point estimate for structure similarity without confidence intervals. Although the statistical significance of differences in GDT_TS between group performances can be tested in CASP where participating groups submitted a number of predictions [4,5], identifying significant differences between individual models with close structural similarity would be challenging for GDT_TS point estimates due to the potential underlying structural flexibility of the modeled proteins.

The flexibility of protein structures could add uncertainty to the atomic positions, which subsequently introduces uncertainty to structure comparison by GDT_TS measure. Currently, CASP models are submitted as sets of coordinates representing accurate atom positions. Although efforts to estimate the certainty of atom positions have been made, such estimations are only accurate for a few top performing models [6]. In addition, the estimated values vary dramatically in scale, which limits their utility in estimation of the uncertainty of atom positions. On the other hand, target structures are snapshots of flexible protein molecules that exist as ensembles of conformational states [7–9]; the atomic fluctuations caused by the dynamic properties of target proteins would contribute to the uncertainty of atom positions in their structures. In our study, we derived the GDT_TS uncertainty from simulated fluctuations of target structures. NMR spectroscopy can reveal the functional dynamics of proteins on a wide range of time scales and is used to generate a structure ensemble of (usually 20) conformations [10]. However, the standard X-ray refinement produces the static structure averaged over time and space for the dynamic ensembles contained in crystals [11]. Although B-factors are thought to reflect the conformational diversity of such ensembles [11], insufficient information about collective motions [12] make it intractable to translate the uncertainty of B-factors into that of GDT_TS scores. To re-capitulate the structural heterogeneous ensembles in the crystal lattices, we performed time-averaged refinement [13] for X-ray datasets to generate structural ensembles for our GDT_TS uncertainty analysis.

Here, we utilize structure ensembles either from NMR deposits or generated by time-average refinements from X-ray structures to determine the uncertainty in GDT_TS scores for CASP models. Our results demonstrate that the time-averaged refinements produced structure ensembles in better agreement with the experimental datasets than the averaged X-ray structures, due to the ability to model anharmonic motions. As GDT_TS increases, its standard deviation (SD) also increases, reaching a maximum of 0.3 and 1.23 for X-ray and NMR structures, respectively. To facilitate score comparisons by the community, we developed a user-friendly web server that produces structure ensembles for NMR and X-ray structures and is accessible at http://prodata.swmed.edu/SEnCS. Our work helps to identify the significance of GDT_TS score differences for structures with high similarity, as well as to provide structure ensembles for estimating SDs of any scores.

## Materials and Methods

### Proteins in Different Crystal Forms

We downloaded the non-redundant pdbaa database from http://dunbrack.fccc.edu/Guoli/culledpdb/pdbaa.gz and identified 1706 protein sequences associated with more than 2 space groups from the pdbaa database. The structures with the highest resolution for each space group were selected as representatives for the GDT_TS calculation. Briefly, representative structures are superimposed by the sequence-independent LGA structural aligner to generate sequence alignments, which in turn were used to produce sequence-dependent GDT_TS scores. We note that protein segments undergoing dramatic conformational changes do not align in the LGA superposition and thus do not contribute to the GDT_TS score calculation.

### Time-Averaged Refinement for X-Ray Structures

We filtered the publically available X-ray structures in CASP9, CASP10, and CASP11 with resolution less than 1.8 Å and obtained 59 high resolution structures. Those structures and their experimental datasets were downloaded from the pdb_redo database [14]. We used the phenix.ensemble_refinement module[13] in the phenix software suit (version 1.9) to perform time-averaged refinement. As the author suggested in the tutorial (http://www.phenix-online.org/documentation/reference/ensemble_refinement.html), we performed simulations with an array of pTLS values: 0, 0.1, 0.2, 0.4, 0.6, 0.8, 0.9, 1.0. We note that the program would automatically adjust the threshold to include at least 63 non-solvent and non-hydrogen atoms; additionally, it would fail if insufficient atoms were included.

### NMR Structure Parsing

33 NMR structures were extracted from CASP9, CASP10, and CASP11 targets and downloaded from the pdb database [15]. To filter flexible regions, we computed the maximum Cα distance deviations of each residue per ensemble. We applied a 3.5 Å maximum Cα threshold, which was used in CASP target processing, to filter flexible residues potentially caused by the insufficient experimental NMR constraints.

### Parameters for Ensembles

We first determined the central model of an ensemble as the structure with the largest sum of pairwise GDT_TS scores to other models in the ensemble. Second, we define selfGDT as the sum of GDT_TS scores comparing the central model to other structures in the ensemble. Finally, we computed the means and standard deviations (SDs) of the selfGDT for all targets, and excluded outlier ensembles (3δ away from the average of the means and SDs). To compare proteins with structures of multiple space groups, we computed the minimal value of all-against-all GDT_TS scores for all models in an ensemble (minGDT) to replace SD as an estimate of ensemble fluctuation. To reduce the sample size of the target ensembles to a number similar to the most prevailing number of crystal forms, we calculated the average minGDT from 1000 random samples of three models from the target ensemble.

### Comparison between Models and Target Structures

Sequence-dependent GDT_TS scores were calculated between the models and the individual structures in an ensemble. GDT_TS mean and standard deviation (SD) were calculated from the population of computed GDT_TS scores for each ensemble. As CASP models include partial structures, we filtered models of NMR structures with less than half of the target sequence length and models of X-ray structures with 100 residues less than the target structures. We binned the SDs by their corresponding means and removed outliers (>3σ) in each bin. When normalizing SDs by the structure flexibility of ensembles, we removed the outlier ensembles using 0.5σ as cutoff for the mean and SDs of selfGDTs, and computed SDs of GDT_TS scores comparing the filtered ensembles against the corresponding models.

### Calculations for GDT_HA and lDDT Scores

The high accuracy version of GDT-based score, i.e. GDT_HA, was computed using LGA, which calculates the percentages of correctly aligned residues under four distance cutoffs: 0.5Å, 1Å, 2Å, and 4Å. Calculating the GDT_HA scores by averaging the correct percentage under these cutoffs, we applied the same pipeline as for the GDT_TS scores to compute the SDs of GDT_HA. We performed linear regression (suppressing the intercept term) for the SDs of GDT_HA and GDT_TS. The R^2^ of the regression model was calculated using Microsoft Excel. Normalization of structure flexibility was performed using a similar procedure as for GDT_TS, substituting 1σ as cutoff for NMR ensembles (the original 0.5σ cutoff excluded all ensembles with GDT_HA greater than 20). The same procedure was also applied to lDDT score calculation, using 0.5σ as the normalization cutoff.

## Results and Discussions

### Generation of Structural Ensembles

CASP targets are primarily determined by X-ray crystallography and sometimes by NMR spectroscopy. NMR structures are deposited as ensembles of multiple conformations indicating the variation due to a combination of protein dynamics and uncertainty in NMR refinement. To generate ensembles indicative of the structural heterogeneity of X-ray structures, we performed time-averaged refinements [13] for crystallographic datasets. Briefly, time-averaged refinement is performed using molecular dynamics simulations with time-averaged constraints on the X-ray dataset. Time-averaged refinement can model anharmonic motions, unlike traditional averaged refinement using B-factors, generating structure ensembles more compatible with the crystallographic data.

**Fig 1:**
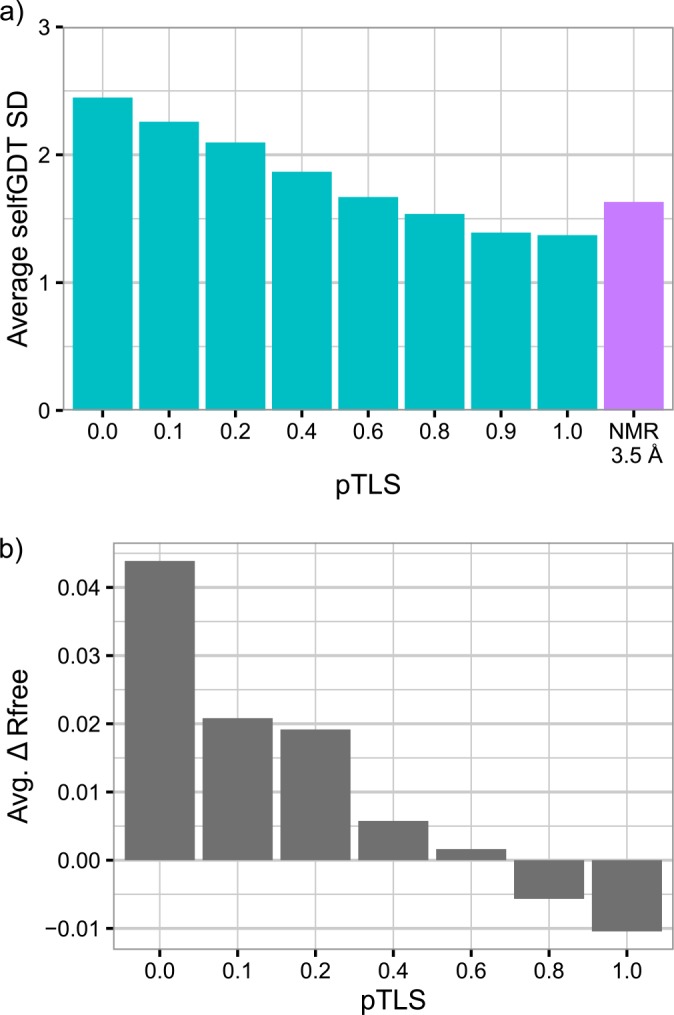
Comparison of structure fluctuations (a) and R-free values (b) for ensembles of different pTLS values. The choice of pTLS influences both the structural variability and the Rfree values of the generated assembles. As pTLS increases, the resulting ensembles show less variability. The structure fluctuations are implied by the SDs of GDT_TS scores calculated between models with an ensemble (selfGDT). NMR (a, purple) ensembles were compared after applying the 3.5Å distance threshold for filtering highly flexible segments. R-free values only improved with respect to experiment when pTLS was greater than 0.6.

In our time-averaged refinement procedure, the global structure flexibility is approximated by the TLS (Translation/Libration/Screw) fitting procedure [16]. This TLS procedure requires a pTLS parameter, which defines the fraction of atoms used in the flexibility approximation and cannot be determined *a priori*. As the authors suggested, we performed simultaneous refinements with an array of pTLS values and observed that the pTLS value controls the amplitude of atomic fluctuations within the produced ensembles. Illustrated by the mean and SDs of selfGDT scores, i.e. the GDT_TS scores comparing models within one ensemble (details in methods), simulations with larger pTLS values produced ensembles of lower structure flexibility exhibiting lower SDs (cyan bars in Fig 1A). More importantly, the time-averaged refinements produced better R-free values only when a sufficient fraction of atoms is included in the flexibility approximation (Fig 1B, R value improvement as 0.01 for pTLS = 1); simulations with pTLS values no more than 0.6 produced worse R-free values (decreasing as much as 0.13) than those of averaged structures and might over-optimize the structure.

**Fig 2:**
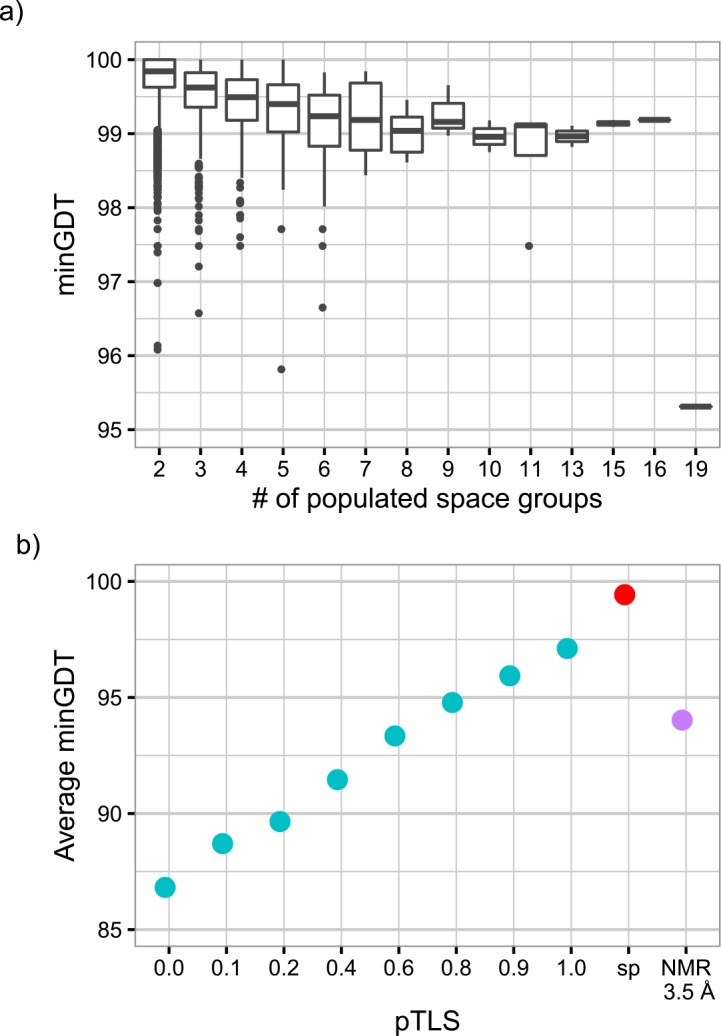
Structure fluctuations of proteins crystallized in different space groups. Structures in different crystal forms serve as a control for the expected structural flexibility. (a) We compared the distribution of minGDTs depending on the number of crystal forms (space groups). (b) The average minGDTs are displayed for the proteins of three space groups (‘SP’, red), time-averaged refinements (cyan), and NMR ensembles (purple). The minGDTs of the latter two (cyan and purple) were computed from resampled ensembles of three random structures. Generated ensembles approached the structural variability seen between different space groups of the same protein at pTLS = 1.

As the choice of pTLS value affects the structure flexibility of the generated ensembles (Fig 1), we chose a pTLS value such that the simulated fluctuations were similar to the expected fluctuations in crystal structures of native proteins. In doing so, we suggest that the observed distribution of GDT_TS scores between members of our simulated ensembles is representative of the true dynamic ensemble of the target protein. To test whether our simulated ensemble was a reasonable model of structural fluctuations, we analyzed cases where the same protein was crystalized in different space groups. Those proteins were experimentally captured in distinct conformational states and were demonstrated to reveal functionally relevant dynamics [17]. A large portion (69%) of these proteins were determined in three distinct space groups (S1 Fig), inhibiting the statistical power of SDs to indicate the structure fluctuations. We used the minimal GDT_TS score (minGDTs) among all scores in an ensemble to indicate the minimal structural similarity of an ensemble. Higher minGDTs implies higher structural similarity and thus lower structure fluctuations. The minGDTs for all proteins lies above 95, with a majority of average minGDT values ranging from 98.9 to 99.5 (Fig 2A). Compared to the majority of such structures (Fig 2B, red dot), time-averaged ensembles exhibit higher fluctuations for all pTLS values (Fig 2B, cyan dots). Therefore, considering both the structure flexibility (indicated by minGDTs) and the compatibility with experimental data (indicated by R-free values), we used the largest possible pTLS value (pTLS = 1).

NMR ensembles showed even higher structure flexibility than X-ray ensembles of pTLS = 1, even when we applied a 3.5Å threshold suggested by the CASP assessors to filter the highly flexible regions (purple bar in Fig 1A). Such differences in structure flexibility were attributed to the discrepancy in environmental influences (such as solvent properties) and experimental interpretation [18]. Most NMR structures are determined in water or organic solvents, whereas proteins in crystallography form a well-ordered crystal lattice with less solvent between protein molecules. When interpreting the experimental data, NMR spectroscopy determines structures with larger allowance for errors from data misinterpretation [19], compared to the high resolution X-ray structures we included (resolution ≤ 1.8Å). Conclusively, consistent with previous studies, our results suggested that NMR ensembles should be more flexible than X-ray ensembles of high resolutions.

### GDT_TS Scores Calculated Using the Ensembles

In CASP evaluations, assessors employed statistical tests, e.g. bootstrapping and Student’s t-test, to identify the top-performing groups [4,5]. However, for comparison between individual models, the lack of uncertainty estimation makes it difficult to distinguish the subtle performance differences between models. Comparisons lacking statistical significance might lead to over-aggressive claims about performance improvement, as small gains could be claimed as performance improvement. To solve this problem, we aimed to estimate the uncertainty of GDT_TS scores from our simulated ensembles to provide confidence intervals for statistical significance.

**Fig 3:**
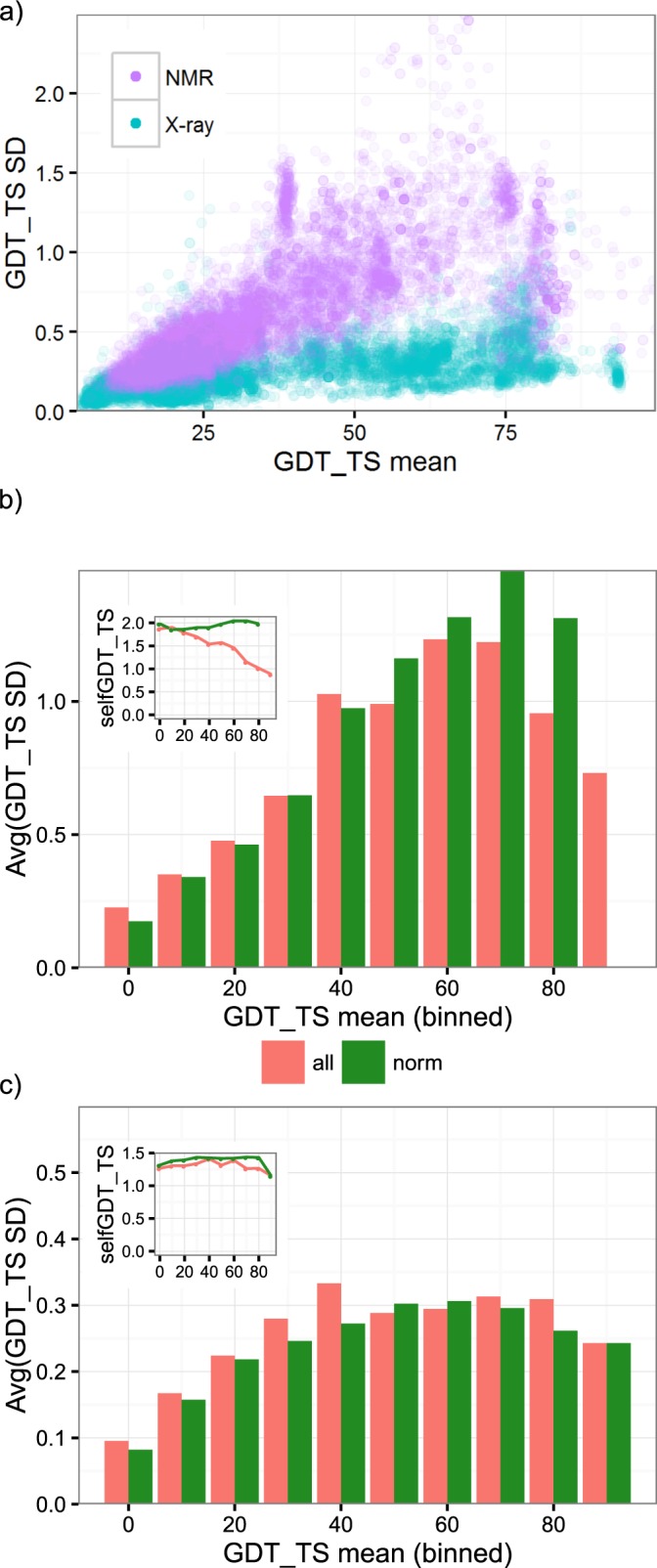
Uncertainty of GDT_TS scores (quantified by the SDs) against the mean of GDT_TS scores. (a) GD_TS scores show a close-to linear relationship between the mean of a GDT_TS score and its standard deviation. The SDs of high scoring NMR models are generally greater than those of X-ray scores in the same regime. We binned SDs for all ensembles before (red bars) and after (green bars) normalization for selfGDTs (shown in insets) for NMR (b) and X-ray (c) ensembles, respectively. Bins are labeled by their left edge. Bins with no models are not shown.

To quantify uncertainty, we computed the standard deviations (SD) of the GDT_TS scores, superimposing models against the generated target ensembles. The mean of such GDT_TS scores would infer the expected value in the canonical comparison between models and a single target structure, as the mean is the most likely value for such GDT_TS scores that follow a normal distribution (refer to S1 File, S2 and S3 Figs for normality test). The SDs in the scatter plots (Fig 3A) exhibited differing scales for X-ray and NMR structures. For further analysis, we binned the SDs by 10 GDT_TS mean and averaged within each bin (Fig 3B and 3C, red bars). The averaged SDs increase with the GDT_TS means for models of low performances, reaching maximum values of 0.3 and 1.23 for X-ray and NMR structures, respectively. The averaged SDs of X-ray ensembles reach the maximum values in bins of smaller GDT_TS mean than NMR ensembles, likely due to the lower structure flexibility of X-ray ensembles. Interestingly, although similar to the maximum values, the average SDs slightly decrease with the GDT_TS mean for high performance models. We also investigated the structure flexibility of ensembles over the bins and found that the models of high GDT_TS scores were predicting the targets of lower structure flexibility; the SDs of GDT_TS comparison within individual ensembles (selfGDT, Fig 3B and 3C inset red lines) decrease for all NMR ensembles and X-ray ensembles of GDT_TS larger than 60. We speculate that such a correlation between the predictability, approximated by the GDT_TS values, of a target and the stability of a protein fold, indicated by the SDs of GDT_TS scores, could be related to the abundance of structure templates. Presumably, lower structural flexibility would facilitate the determination of experimental structures, which could then serve as modeling templates to boost the performance of prediction methods.

To reduce the bias in the distribution of ensemble flexibility over the bins, we further normalized the SDs by filtering a subset of ensembles of similar flexibility (see Methods). The normalized GDT_TS (Fig 3B and 3C green bars) display similar maximums to the raw data (before normalization) for X-ray ensembles, whereas NMR ensembles showed an increased SD as 1.49 due to the reduced sample sizes in high GDT_TS score bins. However, those bins that exhibit the maximum value shift to a higher value, likely due to the exclusion of highly flexible ensembles in lower GDT_TS mean bins. Interestingly, neither NMR nor X-ray ensembles show SDs similar to those of the GDT_TS comparison within individual ensembles (Fig 3B and 3C inset green lines, 1.38 and 1.94 for X-ray and NMR ensembles, respectively), possibly due to superposition optimization. Models of high performance/similarity would potentially superimpose to the conserved core regions of the target structure, leaving the highly flexible loops unaligned and thus reducing the fluctuation of aligned region. On the other hand, low quality models would be aligned over multiple differing regions to individual structures in an ensemble; as a result, the atomic fluctuations in the ensemble are averaged by the superposition optimization.

### Uncertainty of Other CASP Scores

CASP targets are classified into two categories, Template-Based Modeling (TBM) and Free-Modeling (FM), based on the template availability and model performance [20,21]. GDT_TS scores are primarily employed in FM assessment, due to their increased capability to identify high performing models in the presence of short regions with large structural dissimilarities. In the TBM category, the high accuracy version of GDT-based scores, i.e. GDT_HA, was used to better recognize local differences between highly accurate models. Compared to the GDT_TS score, GDT_HA uses stricter distance thresholds for superposition optimization and thus is more sensitive in identifying small improvements in local segments [22]. During CASP11, assessors introduced the superposition-independent Local Distance Difference Test (lDDT) score [23], which is constantly used in Continuous Automated Model EvaluatiOn (CAEMO) [24], to evaluate the local distance difference between structures. In addition to GDT_TS scores, we also evaluated the uncertainty in structure comparison quantified by GDT_HA and lDDT metrics using our generated ensembles.

**Fig 4:**
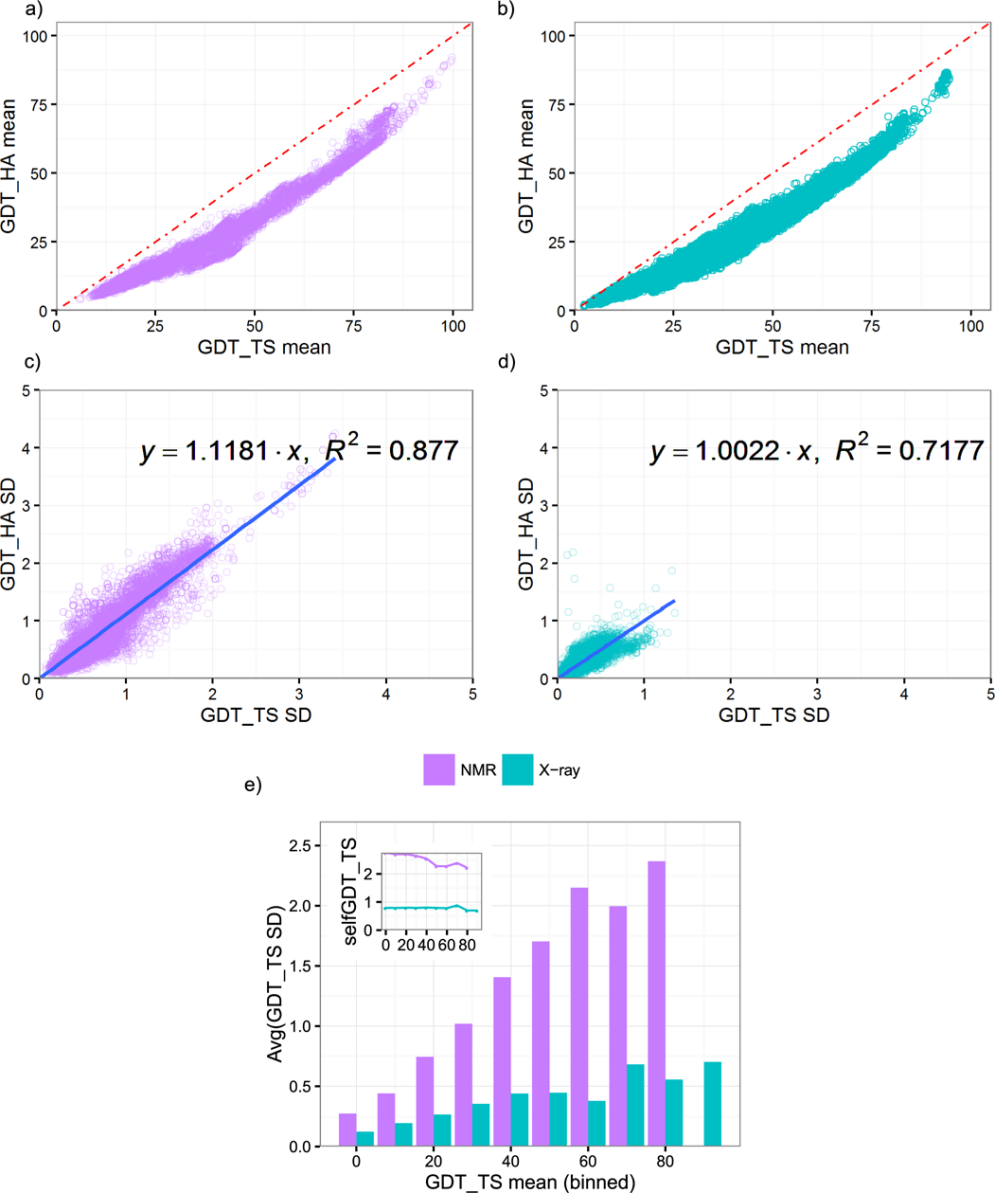
Comparison between GDT_HA and GDT_TS scores on generated ensembles. We compared the relationship between the high-accuracy GDT-based score (GDT_HA) and GDT_TS. Panels a-d display the means and SDs of GDT_HA versus GDT_TS computed from X-ray (cyan) and NMR (purple) models, respectively. Red dashed line denotes the diagonal. Linear regression (blue line) of GDT_HA score SDs with respect to GDT_TS SDs showed a near 1:1 ratio between scores over the observed range. In panel e, binned GDT_HA SDs for filtered ensembles after normalizing the SDs of selfGDTs showed a similar trend to GDT_TS SDs. The X-axis of the panels is labeled by the left edge of the GDT_HA bins. Bins with no models are not shown.

Of the two GDT scores under consideration, GDT_HA is generally 10–20 less than the GDT_TS scores computed from the same models (Fig 4A and 4B), reflecting its higher stringency. The SDs of GDT_HA (shown in Fig 4C and 4D) are correlated with the SDs of GDT_TS scores, with R^2^ of 0.71 and 0.87 for X-ray and NMR ensembles, respectively. Due to increased sensitivity of GDT_HA, we expect that the SDs of GDT_HA would be slightly higher than those of GDT_TS scores; indeed, more than half of GDT_HA scores display higher SDs than those of GDT_TS scores (57.5% for X-ray ensemble and 56.5% for NMR ensembles). GDT_HA, after normalization for structure flexibility, exhibits distributions similar to those of GDT_TS scores (Fig 4E). The SDs of GDT_HA increase with the mean of the scores, reaching a maximum value of 0.45 and 2.36 for X-ray and NMR structures, respectively. Our comparison demonstrates a similar uncertainty distribution for the high accuracy version of GDT-based scores.

**Fig 5:**
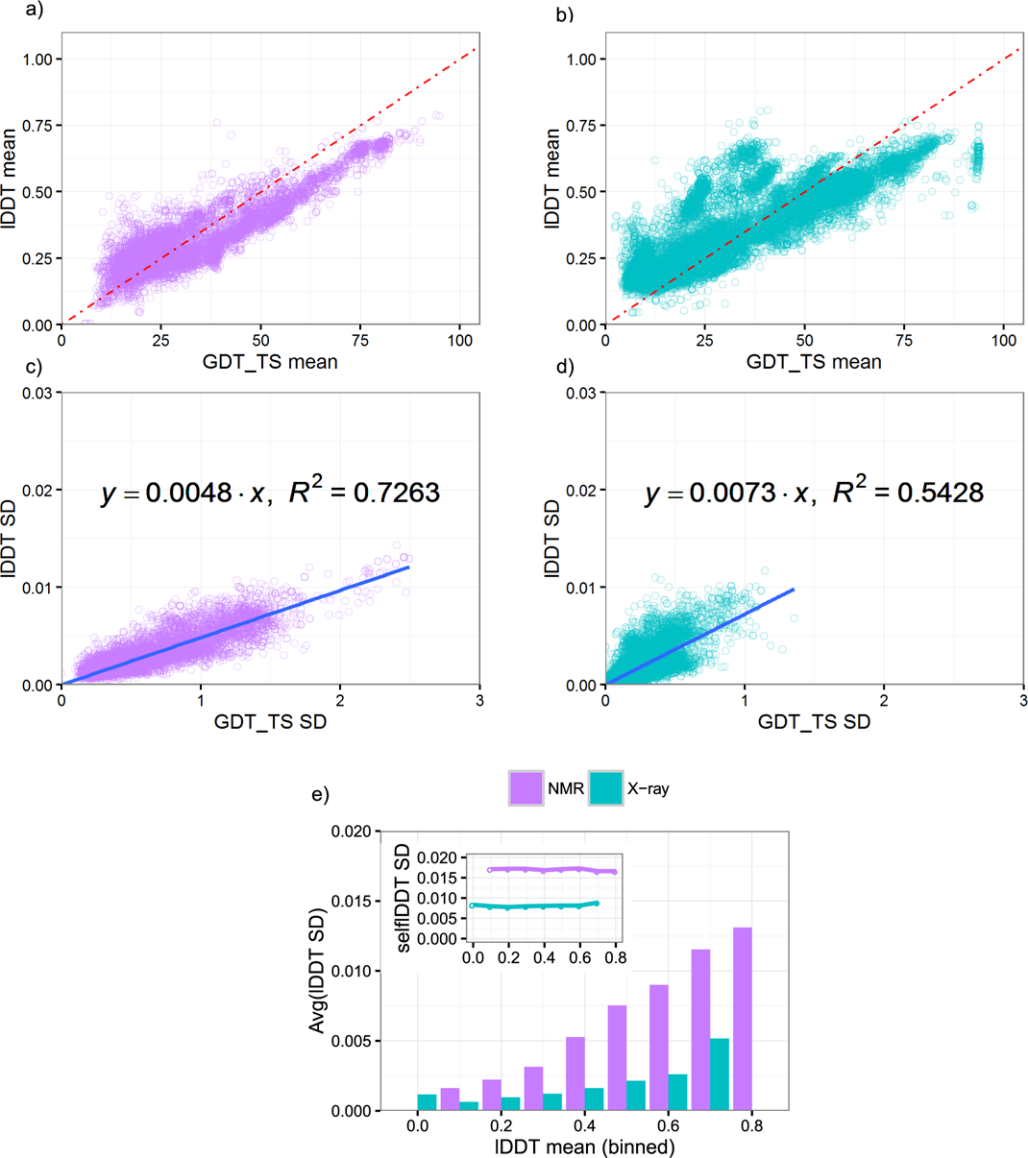
Comparison between lDDT and GDT_TS scores on generated ensembles. Comparisons of mean and SDs were shown for X-ray (cyan) and NMR (purple) ensembles, respectively. Panels a-d display the means and SDs of lDDT versus GDT_TS computed from same models, respectively. Red dash line denote the diagonal. Linear regression (blue line) of lDDT SDs with respect to GDT_TS SDs showed deviations from the 1:1 ratio between scores over the observed range. Panel e illustrated the binned lDDT SDs after normalization, which shows a similar trend to GDT_TS and GDT_HA SDs. The X-axis of the panels is labeled by the left edge of the bin. Bins with 0 models are not shown.

In contrast to the strong correlation between GDT_TS and GDT_HA scores (coefficient as 0.98 for both X-ray and NMR structures), lDDT has a weaker correlation to GDT_TS scores (Fig 5A and 5B, coefficient as 0.82 for X-ray and 0.89 for NMR structures, respectively), which potentially reflects the different evaluation emphasis wherein lDDT scores focus on the preservation of local contacts and GDT_TS highlights the global structure geometry. Consistent with the lower correlations between mean values, the SDs of lDDT and GDT_TS scores have lower R^2^ values of 0.54 and 0.72 for X-ray and NMR structures, respectively (Fig 5C and 5D). Notably, the slope of the linear fits for the SDs showed large deviations from the diagonal (lDDT = 0.01 GDT_TS, 1 GDT_TS score is equivalent to 0.01 lDDT score), especially for NMR structures. Some errors from superposition, which are not included for lDDT scores, could potentially explain the larger SD for GDT_TS scores. The lDDT scores, after normalization for structure flexibility, show similar distributions to those of GDT_TS and GDT_HA scores (Fig 5E). The SDs of lDDT scores increase with the mean of the scores, reaching a maximum value of 0.0051 and 0.0131 for X-ray and NMR structures, respectively. However, due to the lack of high performing models (lDDT>0.8), the observed maximum SDs may not necessarily be the theoretical maximums for lDDT scores, as high performing models could continue the increasing trend for SDs. In conclusion, our study reveals the potential of our generated ensembles in evaluating the uncertainty of any structure similarity metrics.

### Application and Limitations of Estimated Uncertainty in Model Comparison

**Fig 6:**
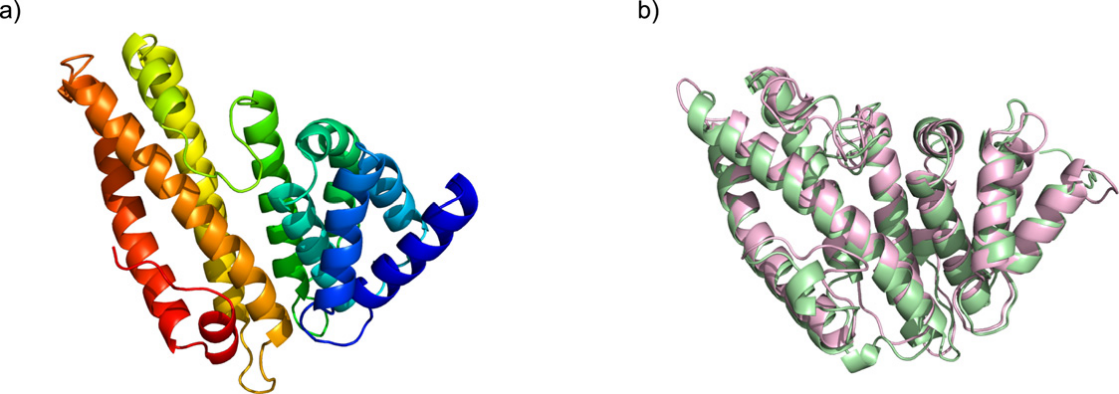
Structure comparison for highly similar models. Two CASP 11 models of TS0839 showed small differences in GDT_TS score, primarily due to differences in their modeled loop regions. Application of our uncertainty estimation reveals that the structural differences between these two models are insignificant and that the models are of similar quality. The model structure colored rainbow was displayed in panel a; the models TS0839TS410_1-D1 (pale-green, GDT_TS = 58.20) and TS839TS117_1-D1 (pink, GDT_TS = 57.72) are superimposed in panel b.

In CASP assessments, the performance significance between groups is established by bootstrap and Student’s t-test [4,5] statistics. However, comparing individual models of close structural similarity can be difficult due to the lack of estimation for the score uncertainty induced by the structural flexibility. Here, we utilized the simulated structural flexibility of prediction targets to provide an estimate of uncertainty potentially underlying an individual point estimate score of a single model structure, which may prevent over-aggressive claims of improved performance. For example, two models from group TS410 and TS117 under target T0839 domain 1 have GDT_TS scores 58.20 and 57.72, respectively. The structural comparison between the models (Fig 6) identified very high similarity between secondary structure elements; however, large structural deviations were observed in the flexible loops connecting those secondary elements. The looped regions from both structures show little structural similarity to the respective regions in the target structure; potentially, the model from TS410 received a higher GDT_TS score due to the incidental overlap of some residues in these loops. By using our estimated uncertainty, the difference between this pair of scores is statistically insignificance under the 95% confidence interval (which requires GDT_TS differs at least 0.6). Therefore, our uncertainty estimation can help identify those models that differ by the random fluctuations in the loop region.

As the GDT_TS scores report the percentage of residues aligned under specified distance cutoff, the length of the structure plays a crucial role in the scale of its variations. For example, one misaligned residue in a protein of 50 residues would cause GDT_TS scores differ by 2, whereas one residue difference in a protein of 200 amino acids would contribute to 0.5 GDT_TS difference. We attempted to study the effect of length on the SDs of GDT_TS scores. Although we can see the tendency for shorter proteins to have larger SDs (S4 Fig), insufficient target numbers for specific protein lengths (S1 Table) hinders the clarification of the quantitative relationship between length and GDT_TS uncertainty. As a single residue misalignment in the shorter protein could potentially create larger score fluctuation that deviates from the most likelihood SDs we concluded, we recommend generating the structure ensembles using our procedure and computing the SDs particularly for short proteins of interest.

### Public Availability of Structural Ensemble Generation

**Fig 7:**
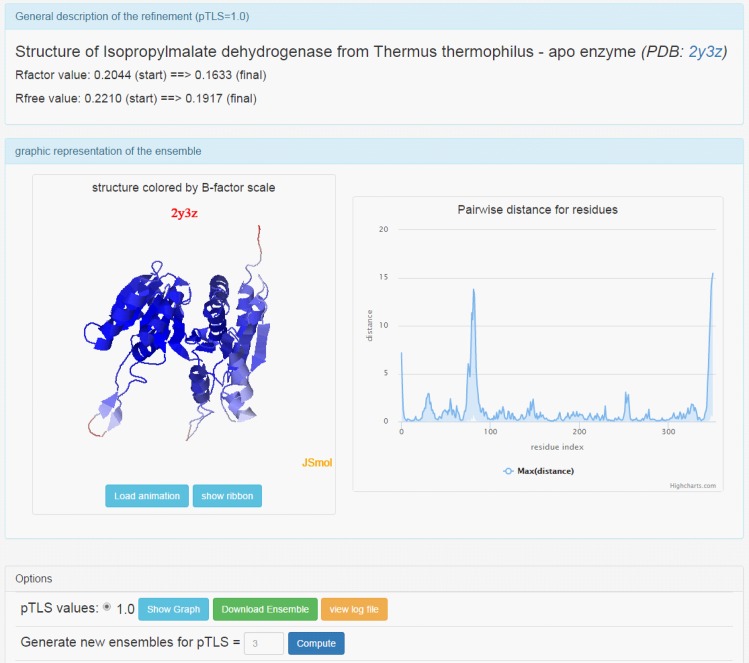
Snapshots of a result page from SEnCS server. This webpage is available via the link: http://prodata.swmed.edu/wenlin/server/enGen/result.php?pdb=2y3z.

To facilitate the SD calculation for any given structure, we implemented our method for generating structure ensembles as a user-friendly web server named SEnCS (Structure Ensemble of Conformational States, available at http://prodata.swmed.edu/wenlin/server/senCS/). The server takes a PDB ID as the input and computes the ensembles based on the type of structures. For NMR structures, it will fetch the ensemble from the PDB database [ref] and process the structure using a 3.5Å threshold to remove highly flexible regions without sufficient NMR constraints. For X-ray structures, it will retrieve the structures and experimental data from the PDB_REDO database [14] and perform time-averaged refinements. By default (fast mode), the time-averaged refinement would use all atoms in flexibility estimation (pTLS = 1) to generate the most conservative ensembles. Alternatively, one can explore an array of atom fractions in flexibility estimation (pTLS value) and generate a series of ensembles (exhaustive mode). The result page (Fig 7) exhibits the structural view of the ensemble in JSmol [25] and the residue-based fluctuation along the protein sequences. The options are available in the result page to vary the distance threshold for NMR ensembles or to compute X-ray ensembles for more user-specified pTLS values. Once the ensemble is generated, users can download them to perform structure comparisons for uncertainty estimation for their scores.

## Conclusions

Our study utilized structural ensembles either from NMR deposits or generated by time-averaged refinement to estimate the uncertainty of GDT_TS scores for CASP models. We quantified the SDs of GDT_TS scores and found that the SDs increase for low GDT_TS models and decrease for high GDT_TS models in our dataset. The X-ray and NMR structures have a maximum SD of 0.3 and 1.23, respectively. Subsequent application of our method to the high accuracy version of GDT-based scores, i.e. GDT_HA, and superposition-independent lDDT scores demonstrates the potential of our procedure to estimate the uncertainty for any other scores. Particularly, GDT_HA produces slightly higher SDs due to the increased sensitivity of GDT_HA. The SDs from lDDT scores are less correlated with those of GDT_TS scores, possibly due to the different dependency of structure superposition. We have also developed a web server that generates structure ensembles for uncertainty estimations. Our work provided generic SDs for estimating confidence intervals of GDT_TS scores, as well as the web server that provides the structure ensembles for any given protein.

## Supporting Information

S1 Fig Statistics for proteins crystalized in different space groups. The number of proteins is shown in logarithmic scale.
(TIF)

S2 Fig Normality test for X-ray ensembles. The plot shows the histogram of R^2^s calculated from X-ray ensembles, with exemplified probability plot against normal distribution in the inset panel.
(TIF)

S3 Fig Normality test for NMR ensembles. (TIF)

S4 Fig The SDs of GDT_TS scores for protein of different lengths against the mean of GDT_TS scores. The structures were binned by 10 GDT_TS and 50 residues. The number in the legend denotes the left edge of the length bin.
(TIF)

S1 File Normality test for GDT_TS scores against target ensembles. (DOCX)

S1 Table Number of targets available for ranges of protein length and GDT_TS scores. The protein lengths are binned by 50 residues and the GDT_TS scores are binned by 10.
(XLSX)
