# Supplementary figures and images for: Estimation of Uncertainties in the Global Distance Test (GDT_TS) for CASP Models
Source: PLoS One. 2016 May 5;11(5):e0154786. doi: 10.1371/journal.pone.0154786 (PMC4858170; doi:10.1371/journal.pone.0154786)

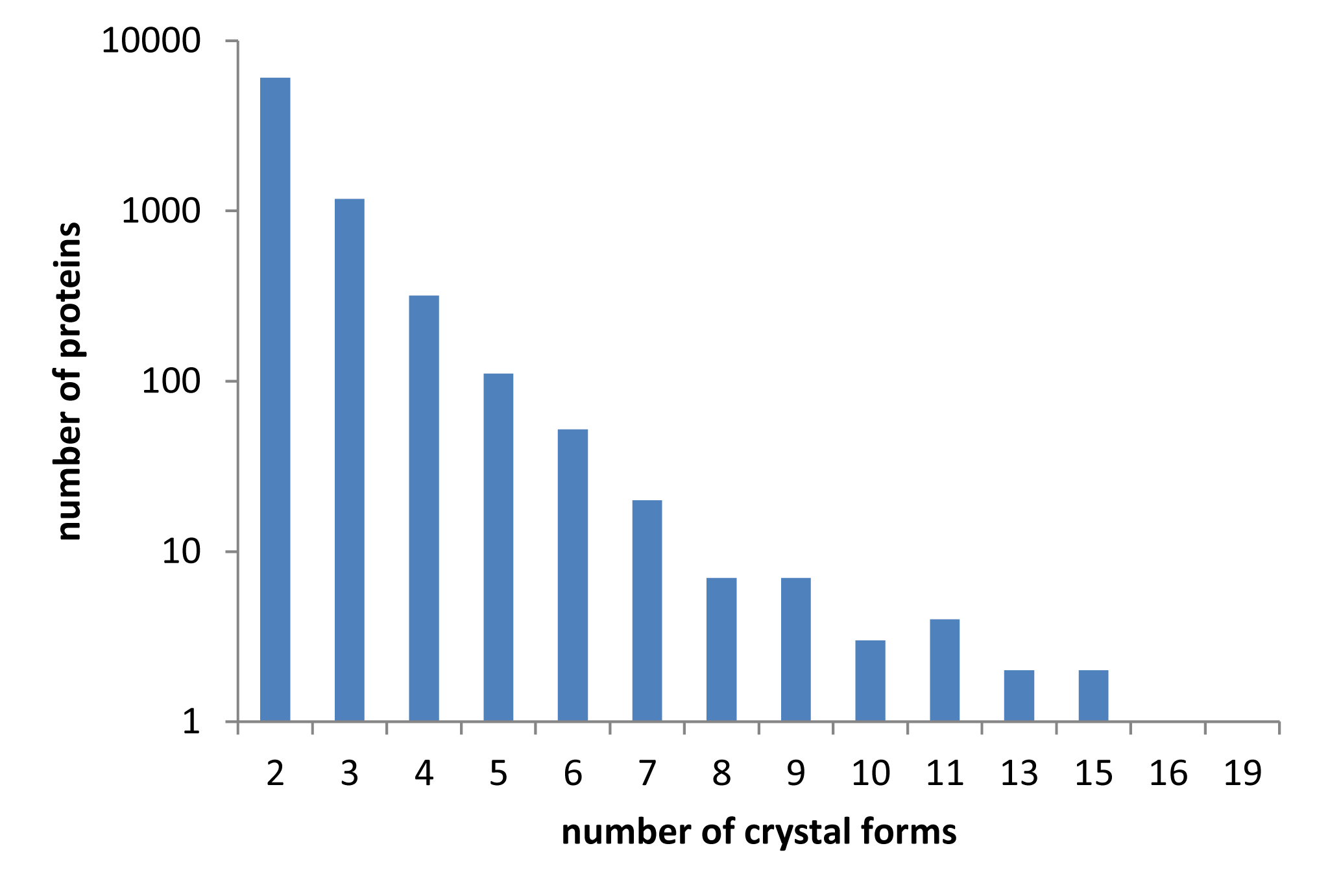

Supplement: S1 Fig — The number of proteins is shown in logarithmic scale. (TIF) [file pone.0154786.s001.tif]

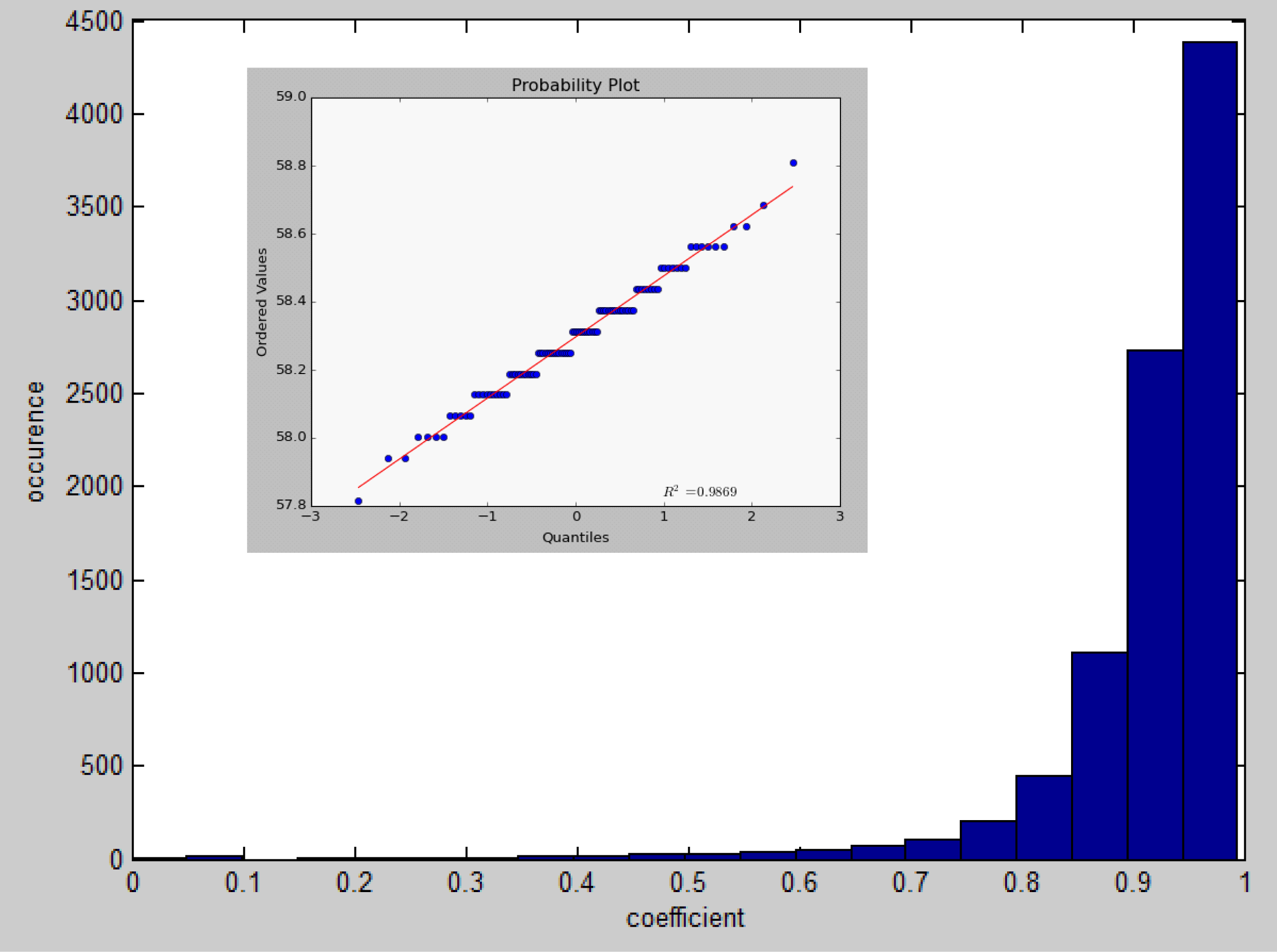

Supplement: S2 Fig — The plot shows the histogram of R2s calculated from X-ray ensembles, with exemplified probability plot against normal distribution in the inset panel. (TIF) [file pone.0154786.s002.tif]

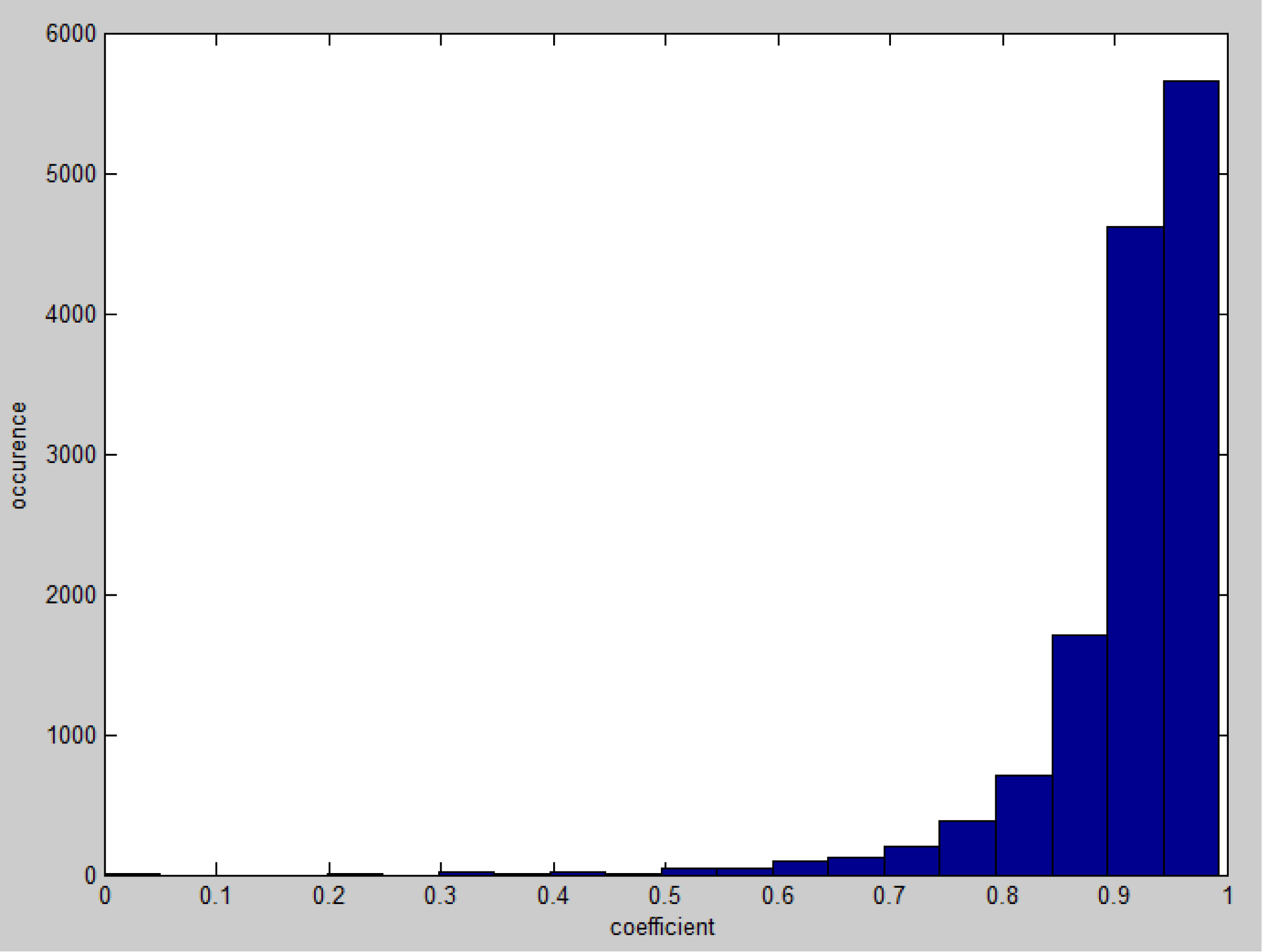

Supplement: S3 Fig — (TIF) [file pone.0154786.s003.tif]

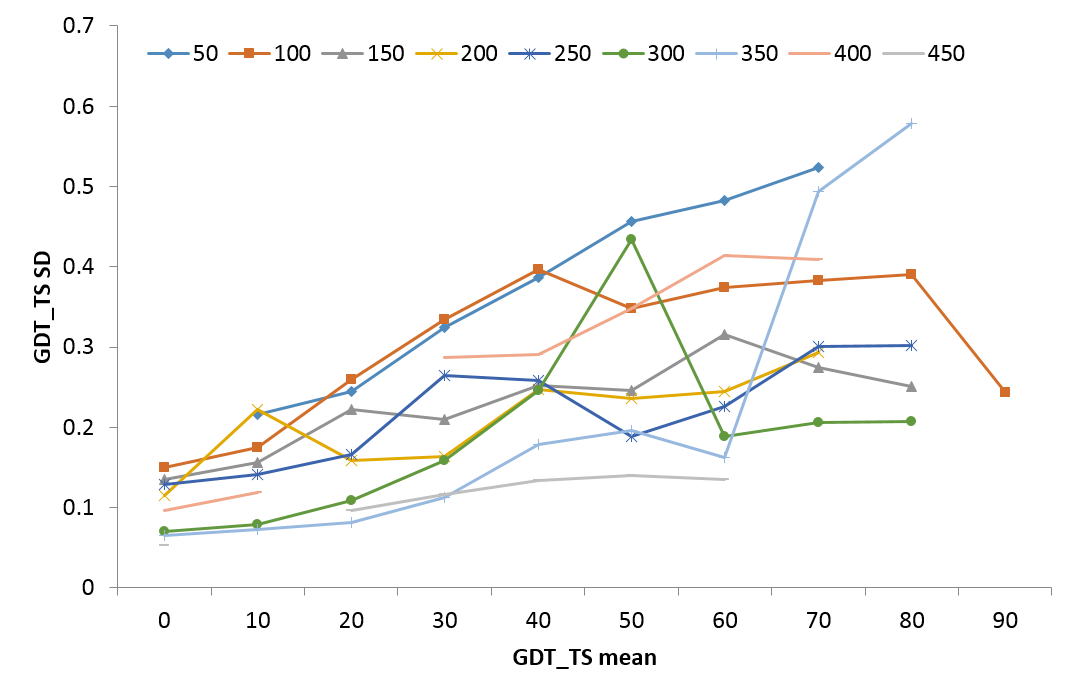

Supplement: S4 Fig — The structures were binned by 10 GDT_TS and 50 residues. The number in the legend denotes the left edge of the length bin. (TIF) [file pone.0154786.s004.tif]
