# Supplementary material for: Estimation of Uncertainties in the Global Distance Test (GDT_TS) for CASP Models
Source: PLoS One. 2016 May 5;11(5):e0154786. doi: 10.1371/journal.pone.0154786 (PMC4858170; doi:10.1371/journal.pone.0154786)
Supplement: S1 File — (DOCX) [file pone.0154786.s005.docx]

**Normality test for GDT scores against target ensembles**

The structure ensembles sample the protein conformational spaces and approximate the structure flexibility of proteins. To determine whether the GDT scores computed from the structures in an ensemble follow a normal distribution, we performed the normality test that measures the goodness of fit to a normal distribution. Such normality test for each model can be visualized as the normal probability plot of GDT scores against the quantiles of an expected normal distribution (example shown in supplemental Fig. 2S, inset panel). A good coefficient of determination (R^2^) to a linear fit would indicate a good approximation by a normal distribution.

To summarize all the normality tests for individual models, we collected the R^2^s from all normality tests and plotted the histogram. We found that the R^2^s include 92.6% of values larger than 0.9 (supplemental Fig. S2), suggesting that GDT scores between one model and its corresponding ensemble can be well-modeled by a normal distribution. We also performed normality test for NMR structures, which shows similar results (supplemental Fig. S3). The established normal distribution would facilitate the usage of standard deviations (STDs) to estimate the confidence intervals. For instances, the 95% confidence interval for a normal distribution would be approximately within 1.96 standard deviation of the mean.
